# Supplementary material for: Gone girl: Richardson's ground squirrel offspring and neighbours are resilient to female removal
Source: R Soc Open Sci. 2019 Sep 4;6(9):190904. doi: 10.1098/rsos.190904 (PMC6774953; doi:10.1098/rsos.190904)
Supplement: Supplementary Table 2. Linear mixed model summary of fecal glucocorticoid metabolite change in offspring following the removal of the mother (or no removal as a control) with a random effect of mother nested in neighbourhood ID [file rsos190904supp2.docx]

Supplementary Table 3. Linear mixed model summary of fecal glucocorticoid metabolite change in offspring in the 3-5 day window following removal of the mother (or no removal, yoked by week to the experimental group as a control) with a random effect of mother nested in neighbourhood ID (n = 36)

|  | Estimate | Std. Error | 95% C. I. | | t | *p* |
| --- | --- | --- | --- | --- | --- | --- |
|  |  |  | Lower | Upper |  |  |
| Intercept (ref: 2014,  Female, no removal) | -221.6 | 2251 | -4634 | 4191 | -0.10 | 0.92 |
| Mother removed | -1.70 | 5.60 | -8.65 | 2.67 | -0.30 | 0.77 |
| Age | 0.02 | 0.05 | -0.08 | 0.14 | 0.32 | 0.75 |
| Sex (male) | -0.80 | 2.50 | -6.34 | 4.70 | -0.31 | 0.76 |
| Year (2015) | 0.10 | 1.10 | -1.07 | 2.42 | 0.10 | 0.92 |
| Removal : Age | <0.001 | 0.20 | -0.07 | 0.28 | 0.00 | 1.0 |
| Removal : Sex (male) | 2.90 | 13.00 | -2.70 | 13.20 | 0.23 | 0.82 |
| Age : Sex (male) | 0.02 | 0.07 | -0.13 | 0.17 | 0.31 | 0.76 |
| Removal : Age : Sex | -0.03 | 0.40 | -0.43 | 0.06 | -0.08 | 0.94 |
