# Supplementary material for: Gone girl: Richardson's ground squirrel offspring and neighbours are resilient to female removal
Source: R Soc Open Sci. 2019 Sep 4;6(9):190904. doi: 10.1098/rsos.190904 (PMC6774953; doi:10.1098/rsos.190904)
Supplement: Supplementary Table 3. Linear mixed model summary of fecal glucocorticoid metabolite change in offspring in the 3–5 day window following removal of the mother (or no removal, yoked by week to the experimental group as a control) with a random effect of mother nested in neighbourhood ID [file rsos190904supp3.docx]

Supplementary Table 2. Linear mixed model summary of fecal glucocorticoid metabolite change in offspring following the removal of the mother (or no removal as a control) with a random effect of mother nested in neighbourhood ID (n = 64)

|  | Estimate | Std. Error | 95% C. I. | | t | *p* |
| --- | --- | --- | --- | --- | --- | --- |
|  |  |  | Lower | Upper |  |  |
| Intercept (ref: 2014,  Female, no removal) | -1.54 | 2.39 | -6.22 | 3.14 | -0.64 | 0.52 |
| Mother removed | -2.99 | 2.89 | -8.65 | 2.67 | -1.04 | 0.31 |
| Age | 0.03 | 0.06 | -0.08 | 0.14 | 0.51 | 0.62 |
| Sex (male) | -0.82 | 2.82 | -6.34 | 4.70 | -0.29 | 0.77 |
| Year (2015) | 0.67 | 0.89 | -1.07 | 2.42 | 0.76 | 0.45 |
| Removal : Age | 0.10 | 0.09 | -0.07 | 0.28 | 1.17 | 0.25 |
| Removal : Sex (male) | 5.25 | 4.06 | -2.70 | 13.20 | 1.29 | 0.21 |
| Age : Sex (male) | 0.02 | 0.08 | -0.13 | 0.17 | 0.26 | 0.80 |
| Removal : Age : Sex | -0.19 | 0.13 | -0.43 | 0.06 | -1.49 | 0.15 |
