# Supplementary material for: Gone girl: Richardson's ground squirrel offspring and neighbours are resilient to female removal
Source: R Soc Open Sci. 2019 Sep 4;6(9):190904. doi: 10.1098/rsos.190904 (PMC6774953; doi:10.1098/rsos.190904)
Supplement: Supplementary Table 4. Binomial GLMM summary of offspring survival following the removal of the mother (or no removal as control) with a random effect of mother nested in neighbourhood ID [file rsos190904supp4.docx]

Supplementary Table 4. Binomial GLMM summary of offspring survival following the removal of the mother (or no removal as control) with a random effect of mother nested in neighbourhood ID (n = 39)

|  | Estimate | Std. Error | 95% C. I. | |  | z | *p* |
| --- | --- | --- | --- | --- | --- | --- | --- |
|  |  |  | Lower | Upper |  |  |  |
| Intercept (ref: 2014, no removal) | -1.39 | 1.12 | -1.64 | 2.25 |  | -1.24 | 0.22 |
| Mother removed | 0.13 | 1.38 | -3.34 | 1.53 |  | 0.10 | 0.92 |
| Year (2015) | -0.12 | 1.36 | -2.87 | 1.70 |  | -0.09 | 0.93 |
| Removal : Year | 0.78 | 1.68 | -2.16 | 3.70 |  | 0.47 | 0.64 |
