# Supplementary material for: Gone girl: Richardson's ground squirrel offspring and neighbours are resilient to female removal
Source: R Soc Open Sci. 2019 Sep 4;6(9):190904. doi: 10.1098/rsos.190904 (PMC6774953; doi:10.1098/rsos.190904)
Supplement: Supplementary Table 5. Linear mixed model summary of fecal glucocorticoid metabolite change in neighbours in the 3–5 day window following kin and non-kin neighbour removal (or no removal as control), with a random effect of neighbour nested in neighbourhood ID [file rsos190904supp5.docx]

Supplementary Table 5. Linear mixed model summary of fecal glucocorticoid metabolite change in neighbours in the 3-5 day window following kin and non-kin neighbour removal (or no removal as control), with a random effect of neighbour nested in neighbourhood ID (n = 83)

|  | Estimate | Std. Error | 95% C. I. | |  | t | *p* |
| --- | --- | --- | --- | --- | --- | --- | --- |
|  |  |  | Lower | Upper |  |  |  |
| Intercept (ref: 2014, no removal, 0 relatedness) | 1.55 | 1.36 | -1.12 | 4.22 |  | 1.14 | 0.27 |
| Female removed | 1.14 | 2.35 | -3.47 | 5.75 |  | 0.48 | 0.63 |
| Relatedness coefficient to neighbour | -0.70 | 4.64 | -9.79 | 8.40 |  | -0.15 | 0.88 |
| Year (2015) | -0.49 | 1.64 | -3.71 | 2.72 |  | -0.30 | 0.76 |
| Removal : Relatedness | -10.90 | 11.72 | -33.87 | 12.08 |  | -0.93 | 0.36 |
| Removal : Year (2015) | -2.50 | 3.08 | -8.53 | 3.53 |  | -0.81 | 0.42 |
| Relatedness : Year (2015) | -0.92 | 5.94 | -12.57 | 10.73 |  | -0.16 | 0.88 |
| Removal : Relatedness : Year | 15.10 | 14.40 | -13.22 | 43.32 |  | 1.04 | 0.30 |
