# Supplementary material for: Gone girl: Richardson's ground squirrel offspring and neighbours are resilient to female removal
Source: R Soc Open Sci. 2019 Sep 4;6(9):190904. doi: 10.1098/rsos.190904 (PMC6774953; doi:10.1098/rsos.190904)
Supplement: Supplementary Table 6. Binomial GLMM summary of fixed effects predicting neighbour survival following kin or non-kin neighbour removal (or no removal as control), with a random effect of neighbour nested in neighbourhood ID [file rsos190904supp6.docx]

Supplementary Table 6. Binomial GLMM summary of fixed effects predicting neighbour survival following kin or non-kin neighbour removal (or no removal as control), with a random effect of neighbour nested in neighbourhood ID (n = 67)

|  | Estimate | Std. Error | 95% C. I. | |  | z | *p* |
| --- | --- | --- | --- | --- | --- | --- | --- |
|  |  |  | Lower | Upper |  |  |  |
| Intercept (ref: 2014, 0 proportion removed, no kin removed) | -0.25 | 0.52 | -1.27 | 0.76 |  | -0.49 | 0.63 |
| Proportion neighbours removed | -1.20 | 1.35 | -3.84 | 1.44 |  | -0.89 | 0.37 |
| Kin removal (binary; Y) | 0.99 | 0.83 | -0.62 | 2.61 |  | 1.20 | 0.23 |
| Year (2015) | 0.79 | 0.58 | -0.35 | 1.93 |  | 1.35 | 0.18 |
| Proportion removed : kin removal | -1.59 | 2.72 | -6.92 | 3.74 |  | -0.59 | 0.56 |
